# Editorial: Synthesis, functionalization, and clinical translation of pharmaceutical biomaterials

**DOI:** 10.3389/fbioe.2022.1096511

**Published:** 2022-12-09

**Authors:** Huicong Zhou, Shixian Lv, Mingqiang Li, Jianxun Ding

**Affiliations:** ^1^ School of Materials Science and Engineering, Peking University, Beijing, China; ^2^ Laboratory of Biomaterials and Translational Medicine, Center for Nanomedicine, The Third Affiliated Hospital, Sun Yat-sen University, Guangzhou, China; ^3^ Key Laboratory of Polymer Ecomaterials, Changchun Institute of Applied Chemistry, Chinese Academy of Sciences, Changchun, China

**Keywords:** biomaterial, drug delivery, biotechnology, pharmaceutics, disease assessment

In recent decades, biomaterials played a pivotal role in the efficient and targeted delivery of therapeutic agents, biomedical manipulation, and disease assessment. Meanwhile, advances in synthesis and functionalization technologies of pharmaceutical biomaterials continuously provide unique clinical translation strategies.

This invited Research Topic consisted of 7 articles, including 4 original research articles and 3 review articles, contributed by 42 researchers worldwide (Total views: 7.4 k by 6 December 2022). These review articles cover Research Topics discussing nanomaterials and the precise detection and treatment of tumors. One of these reviews provided a systematic summary of all aspects of nano-vesicles as drug delivery vectors for cancer therapy: characteristics of polymeric nanovesicles, synthesis of polymeric nanovesicle, and recent progress in applying polymeric nanovesicles in antitumor drug delivery (Li et al.). The specialized structures and properties of polymeric nanovesicles exhibited good stability and permeability, easy functionalization, and smart stimulus responsiveness, which made them one of the most promising supramolecular structures for potential applications in delivering drugs, genes, and other therapeutic substances. The other reviews discussed various biomaterials applied to improve pharmacological efficacy and biological safety, such as peptides, nanorobots, or nanoparticles for tumor infarction therapy (Tong et al.), and aptamer nanomaterials as targeted theranostic platforms for ovarian cancers (Zhao et al.). Using nanomaterials as carriers to combine therapeutic agents overcame the shortcomings of a single agent, which showed great potential in clinical translation.

In this Research Topic, all the original studies showed critical aspects of pharmaceutical biomaterials on their synthesis, functionalization, and potential clinical translation. Emerging pharmaceutical biomaterials were widely used in drug delivery and disease assessment. For instance, to improve therapeutic efficacies, combination therapy using multiple drug agents is necessary, and the employment of biomaterials as drug delivery systems ensures drug delivery efficiency and biological safety. For instance, a reduction-responsive cross-linked nanogel methoxy poly(ethylene glycol)-poly(L-phenylalanine-*co*-L-cystine) (mPEG-(LP-*co*-LC)) was synthesized using the ring-opening reaction of amino acid *N*-carboxylanhydride (Liu Y. et al.). Olaparib and doxorubicin were co-loaded by the nanogel, and the drug could be quickly released to target cancer cells under the stimulation of a high glutathione concentration in the cancer cells. Compared with free drug and single drug-loaded nanogel, the dual-drug-co-loaded nanogel exhibited the best anti-cancer efficacy and demonstrated excellent biological safety. Likewise, the biomimetic nanoparticle Poly(lactic-*co*-glycolic acid)-dutasteride/siAR@membrane of human dermal papilla cells (PLGA-DUT/siAR@DPCM) simultaneously encapsulating dutasteride and siRNA was used for the synergistic treatment of androgenic alopecia *via* the suppression of 5α-reductase and knockdown of androgen receptor (Chen et al.). The nanoparticle was coated with the biomimetic membrane of human dermal papilla cells (hDPCs), enabling the nanoparticles to have better hDPC penetration and higher delivery efficiency.

Apart from improving the therapeutic outcomes as drug delivery vehicles, some advanced biomaterials also manipulate cell behaviors. For instance, metformin carbon nanodot (MCD) was synthesized to promote odontoblastic differentiation of dental pulp stem cells (DPSCs) by the pathway of autophagy to achieve functional pulp regeneration (Lu et al.). MCD was capable of activating autophagy and enhancing the odontogenic differentiation of human dental pulp stem cells (hDPSCs) by upregulating odontoblast gene markers (DMP1, DSPP, RUNX2, and SP7) and expression of proteins (DSPP and DMP1).

Lastly, although nanoparticles have been widely studied as drug nano carriers and therapeutic agents, the evaluation of biological distribution of nanoparticles in the tumor microenvironments (TMEs) is still necessary. For example, using vibratome sectioning of tumors provided a method to evaluate the interactions between nanoparticles and the TMEs *ex vivo* (Liu S. et al.). The cell morphology and activity were maintained in the vibratome sections, and the distribution of nanocarriers in the vibratome tumor sections was similar to that observed *in vivo*. *Ex vivo* analysis of tumor tissue slices provided a more convenient and stable method for elucidating the distribution of nanocarriers in the TMEs.

Overall, the current Research Topic shows the broad applicability of pharmaceutical biomaterials in precisely drug delivery and disease evaluation. Growing evidence suggests that these prospective studies will establish a connection to clinical translation and support the development of biomaterials, bioengineering, biotechnology, and pharmaceutics.

